# An End-to-End Integrated Clinical and CT-Based Radiomics Nomogram for Predicting Disease Severity and Need for Ventilator Support in COVID-19 Patients: A Large Multisite Retrospective Study

**DOI:** 10.3389/fradi.2022.781536

**Published:** 2022-04-08

**Authors:** Pranjal Vaidya, Mehdi Alilou, Amogh Hiremath, Amit Gupta, Kaustav Bera, Jennifer Furin, Keith Armitage, Robert Gilkeson, Lei Yuan, Pingfu Fu, Cheng Lu, Mengyao Ji, Anant Madabhushi

**Affiliations:** ^1^Department of Biomedical Engineering, Case Western Reserve University, Cleveland, OH, United States; ^2^Department of Radiology, University Hospitals Cleveland Medical Center, Cleveland, OH, United States; ^3^Department of Internal Medicine, Maimonides Medical Center, Brooklyn, NY, United States; ^4^Department of Infectious Diseases, University Hospitals Cleveland Medical Center, Cleveland, OH, United States; ^5^Department of Information Center, Renmin Hospital of Wuhan University, Wuhan, China; ^6^Department of Population and Quantitative Health Sciences, Case Western Reserve University, Cleveland, OH, United States; ^7^Department of Gastroenterology, Renmin Hospital of Wuhan University, Wuhan, China; ^8^Louis Stokes Cleveland Veterans Administration Medical Center, Cleveland, OH, United States

**Keywords:** COVID-19, radiomics, nomogram, prognosis, severity, peritumoral radiomics

## Abstract

**Objective:**

The disease COVID-19 has caused a widespread global pandemic with ~3. 93 million deaths worldwide. In this work, we present three models—radiomics (M_RM_), clinical (M_CM_), and combined clinical–radiomics (M_RCM_) nomogram to predict COVID-19-positive patients who will end up needing invasive mechanical ventilation from the baseline CT scans.

**Methods:**

We performed a retrospective multicohort study of individuals with COVID-19-positive findings for a total of 897 patients from two different institutions (Renmin Hospital of Wuhan University, D_1_ = 787, and University Hospitals, US D_2_ = 110). The patients from institution-1 were divided into 60% training, ${\text{D}}_{1}^{\text{T}}$ (*N* = 473), and 40% test set ${\text{D}}_{1}^{\text{V}}$ (*N* = 314). The patients from institution-2 were used for an independent validation test set ${\text{D}}_{2}^{\text{V}}$ (*N* = 110). A U-Net-based neural network (CNN) was trained to automatically segment out the COVID consolidation regions on the CT scans. The segmented regions from the CT scans were used for extracting first- and higher-order radiomic textural features. The top radiomic and clinical features were selected using the least absolute shrinkage and selection operator (LASSO) with an optimal binomial regression model within ${\text{D}}_{1}^{\text{T}}$.

**Results:**

The three out of the top five features identified using ${\text{D}}_{1}^{\text{T}}$ were higher-order textural features (GLCM, GLRLM, GLSZM), whereas the last two features included the total absolute infection size on the CT scan and the total intensity of the COVID consolidations. The radiomics model (M_RM_) was constructed using the radiomic score built using the coefficients obtained from the LASSO logistic model used within the linear regression (LR) classifier. The M_RM_ yielded an area under the receiver operating characteristic curve (AUC) of 0.754 (0.709–0.799) on ${\text{D}}_{1}^{\text{T}}$, 0.836 on ${\text{D}}_{1}^{\text{V}}$, and 0.748 ${\text{D}}_{2}^{\text{V}}$. The top prognostic clinical factors identified in the analysis were dehydrogenase (LDH), age, and albumin (ALB). The clinical model had an AUC of 0.784 (0.743–0.825) on ${\text{D}}_{1}^{\text{T}}$, 0.813 on ${\text{D}}_{1}^{\text{V}}$, and 0.688 on ${\text{D}}_{2}^{\text{V}}$. Finally, the combined model, M_RCM_ integrating radiomic score, age, LDH and ALB, yielded an AUC of 0.814 (0.774–0.853) on ${\text{D}}_{1}^{\text{T}}$, 0.847 on ${\text{D}}_{1}^{\text{V}}$, and 0.771 on ${\text{D}}_{2}^{\text{V}}$. The M_RCM_ had an overall improvement in the performance of ~5.85% (${\text{D}}_{1}^{\text{T}}$: *p* = 0.0031; ${\text{D}}_{1}^{\text{V}}$ *p* = 0.0165; ${\text{D}}_{2}^{\text{V}}$: *p* = 0.0369) over M_CM_.

**Conclusion:**

The novel integrated imaging and clinical model (M_RCM_) outperformed both models (M_RM_) and (M_CM_). Our results across multiple sites suggest that the integrated nomogram could help identify COVID-19 patients with more severe disease phenotype and potentially require mechanical ventilation.

## Introduction

The coronavirus disease 2019 (COVID-19), caused by severe acute respiratory syndrome 2 (SARS-CoV-2), is an ongoing global pandemic with over 3.93 million deaths and 181 million total diagnosed cases worldwide so far (1–3). The new COVID-19 delta variant, recently diagnosed and spreading across the world, has the ability to cause very dense outbreaks (4, 5).

The majority of COVID-19 patients present with mild disease to an outpatient clinic or *via* telehealth with minor clinical symptoms. A lesser proportion of the patients develop moderate to severe disease with significant pulmonary dysfunction or damage as evidenced by signs of hypoxemia and moderate to severe dyspnea (2). According to one study, ~20% of diagnosed COVID-19 cases have severe or critical diseases, and about 8% of them require intensive care management with or without mechanical ventilation (6). If we can diagnose this high-risk population at the earliest stages, it will likely allow for optimal resource management and individualized treatment planning (7, 8).

Imaging plays an essential role in the management of COVID-19 patients, with chest CT being the preferred modality for these patients (9). However, despite the high sensitivity of chest CT, the reported specificity is quite low at about 25–33%, which is due to considerable overlap in CT imaging features of COVID-19 and other viral types of pneumonia (10). This, coupled with other challenges, such as transmission risk to uninfected health care workers and other patients, consumption of PPE, and need for cleaning and downtime of radiology equipment in resource-constrained environments, has led to the recommendation by multiple professional societies against usage of CT as a routine screening test for COVID-19 but reserved for only selected clinical scenarios (11).

Furthermore, a variety of prediction models have been reported for diagnosing and prognosticating COVID-19, including a combination of clinical and lab data as well as imaging features (12–16). According to a systematic review, flu-like symptoms and neutrophil count are more predictive in diagnostic models, while comorbidities, sex, C reactive protein, and serum creatinine levels are the frequently reported prognostic factors (17). Most of the AI analysis has focused on chest x-rays (CXRs) (1, 2), though more recently, more and more works on AI for CT scans have also been published. In this work, our focus has been solely on CT scans, and especially machine learning-based models. However, many of the proposed models are poorly reported and are at high risk of bias, and at present, it is not recommended to use any of the reported prediction models for use in clinical practice (17).

Therefore, there is an unmet need to develop non-invasive tools, preferably based on existing imaging techniques and available clinical parameters, that can help prospectively identify patients at higher risk for developing severe disease phenotype. The ability to identify these patients who will probably need mechanical ventilation and develop severe symptoms will allow us for optimal use of existing precious resources.

In the past few years, high-throughput computer extracted features from the radiographic images (radiomics) has been useful for a variety of diagnostic, prognostic, and predictive applications across several cancers as well as other diseases (18–20). These features are known to capture the underlying tissue morphology and characteristics, which are not visually apparent to the naked eyes (21, 22). Within the COVID-19 space, radiomics has been used for various applications. Radiomics has been successful in differentiating COVID-19 patients from other pneumonia cases (diagnostic), as well as has shown application for predicting the severity of COVID-19 patients (prognostic). Table 1 in the Appendix 1 shows the studies from December 2019 to December 2020 looking at various machine-learning radiomic-based models for diagnostics as well as prognostic applications (13–15, 23–28).

**Table 1:** Patient characteristics.

	**Renmin Hospital (Wuhan, China)—D**_**1**_		**University Hospitals (Cleveland, US)—D_**2**_**
	**Train**	**Test**	
Age median (IQR)	59 (46–67)	60 (48–69)	62 (20–94)
**Ventilator**			
Yes (%)	267 (62.09%)	154	28
No (%)	163	137	85
**Laboratory findings**			
Lactate dehydrogenase (U/L)	280.7 (126–936)	271.8 (108–1,039)	348.5 (127–919)
Albumin (g/L)	37.29 (22.7–49)	38.48 (24.6–50.6)	36 (25.0–44.0)
**Radiomics**			
Radiomic score median (IQR)	2.99 (2.57–3.47)	2.98 (2.46–3.52)	3.51 (3.184.02)

In this work, we aim to combine the clinical and laboratory parameters with imaging data to build an accurate and easy-to-use nomogram to predict the need for mechanical ventilation for COVID-19 patients. The imaging data includes radiomic features extracted from the regions corresponding to COVID consolidation on CT scans; these regions of consolidation were automatically segmented using the U-Net-based model, making the whole end-to-end pipeline completely automated. Our model has been validated on roughly ~1,000 patients from two different institutions making this one of the largest radiomic-based prognosis predictions for COVID-19 studies to date.

## Materials and Methods

### Patients

**Figure 1:**
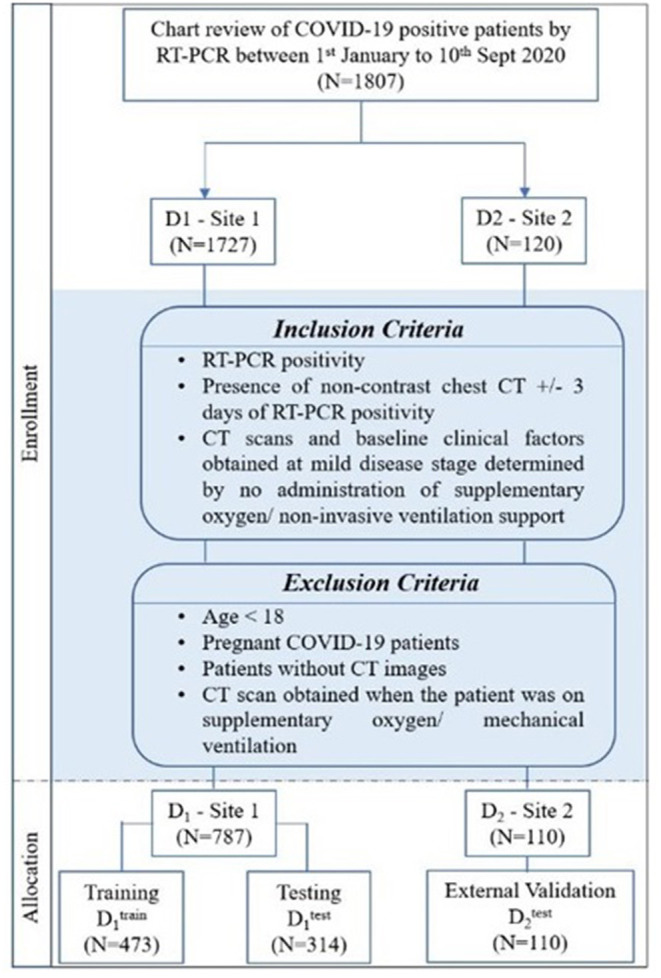
Patient selection criteria and dataset distribution.

The Institutional Review Board Committee approved the retrospective chart review study of record at the University Hospitals, Cleveland (STUDY20200463), and the Renmin Hospital of Wuhan University (ethics number: V1.0; IRB number 2020KS02010). The need for written consent was waived. Following the inclusion and exclusion criteria, the study included D_1_ (*N* = 787) patients from the hospital of Wuhan University, Hubei General Hospital, and D_2_ (*N* = 110) patients from University Hospitals, Cleveland. The details regarding the inclusion–exclusion criteria and patient flowchart are mentioned in Figure 1.

Stratified random sampling was performed to split the data from institution-1 into 60% training ${\text{D}}_{1}^{\text{T}}$ (*N* = 473) and 40% testing ${\text{D}}_{1}^{\text{V}}$ (*N* = 314). While randomly dividing the data, the COVID patients being on the ventilator were kept approximately similar within training and testing cohorts (The training cohort had ~64% of the COVID patients being on ventilator, whereas ~55% of the COVID patients did not use the ventilator. Similarly, the testing set had ~36% of the COVID patients who used the ventilator, and ~45% of the COVID patients did not use the ventilator).

The patients from institution-2 were used for independent external validation ${\text{D}}_{2}^{\text{V}}$ (*N* = 110). The patients were acquired by following the chart review for patients who were seen between January and September 2020.

### Radiomic Feature Analysis

#### Detection and Segmentation of Lung Lesions

An expert radiologist with 14 years of experience delineated ground-glass (GGO) and consolidation regions on a subset of ${\text{D}}_{1}^{\text{T}}$ [${\text{D}}_{\text{UNET}}^{\text{T}}$ *N* = 88 (training cohort) and ${\text{D}}_{\text{UNET}}^{\text{V}}$ *N* = 96 (validation cohort)]. The UNET-based model to segment the COVID consolidations on CT scans was trained within a threefold cross-validation setting using ${\text{D}}_{\text{UNET}}^{\text{T}}$, and the performance was validated on ${\text{D}}_{\text{UNET}}^{\text{V}}$. A CNN with U-Net architecture was employed to segment out ground-glass opacities (GGOs) and consolidations in the lung region on the baseline chest CT scans (29). An automatic lung segmentation method utilizing a watershed transform was used to segment out and crop the CT volume around the regions of the lung (30). Each 2D slice of the cropped volume was resized to a size of 256 by 320. Furthermore, the 2D slice was vertically divided into two parts dividing the right and left lung regions (input size: 256 by160), and parts of the lung region (right, left) were given as separate inputs (input size: 256 by160). The two vertical slices from each 2D input were used as inputs to the UNET model to segment COVID consolidations.

Appendix 1 explains the architectural diagram of the 2D U-Net used for segmentation of GGOs and consolidations.

#### Radiomic Feature Extraction

After automatic segmentation of lung volume, all the scans were resampled to 0.75 mm in the x- and y-directions and simultaneously added a uniform slice thickness of 5 mm to reduce the impact of different equipment and scanning parameters. The total infection size was calculated by calculating the volume of the COVID consolidations annotated using the U-Net model. These consolidations were termed as COVID regions. Next, a total of 187 radiomic features were extracted from annotated CT scans. These features included 37 first-order features and 150 higher-order textural features. The textural features included the gray-level co-occurrence matrix (GLCM), gray-level size zone matrix (GLSZM), gray-level run length matrix (GLRLM), neighboring gray tone difference matrix (NGTDM), and gray level dependence matrix (GLDM).

Appendix 1 summarizes all the extracted features. These features capture textural patterns of COVID consolidations that are not apparent with the naked eye and could potentially help describe the heterogeneity of these regions.

The top predictive radiomic features from the training cohort ${\text{D}}_{1}^{\text{T}}$ were selected using the least absolute shrinkage and selection operator (LASSO) feature selection algorithm (31). These features were further used for constructing a continuous radiomic risk score using the weighted sum of their LASSO coefficients. The radiomics model (M_RM_) was constructed using this developed radiomic risk score.

### Clinical Feature Analysis

A total of 20 clinical variables and laboratory parameters were included in the analysis, as explained in Appendix 1. Specifically, these features included patients' age and laboratory parameters, such as albumin (ALB), lymphocytes, WBCs, etc. Previous studies show a high correlation of these clinical variables with the patient being on the ventilator when admitted to a hospital (32, 33).

A total of 545 cases out of 897 had all the clinical variables available. The total missing rate of the clinical variables was 39.34%. To make use of all the available data, the missing clinical values were imputed by the mean values of available clinical entities from ${\text{D}}_{1}^{\text{T}}$. For an external validation set, the missing values were replaced by the mean obtained from the complete cases of the same cohort.

Similar to radiomics analysis, the most prognostic clinical variables were selected from the training cohort ${\text{D}}_{1}^{\text{T}}$ using LASSO analysis (31) and used within the logistic regression model for predicting the need for ventilators in COVID-19 patients (clinical model: M_CM_).

### Statistical Analysis

**Figure 2:**
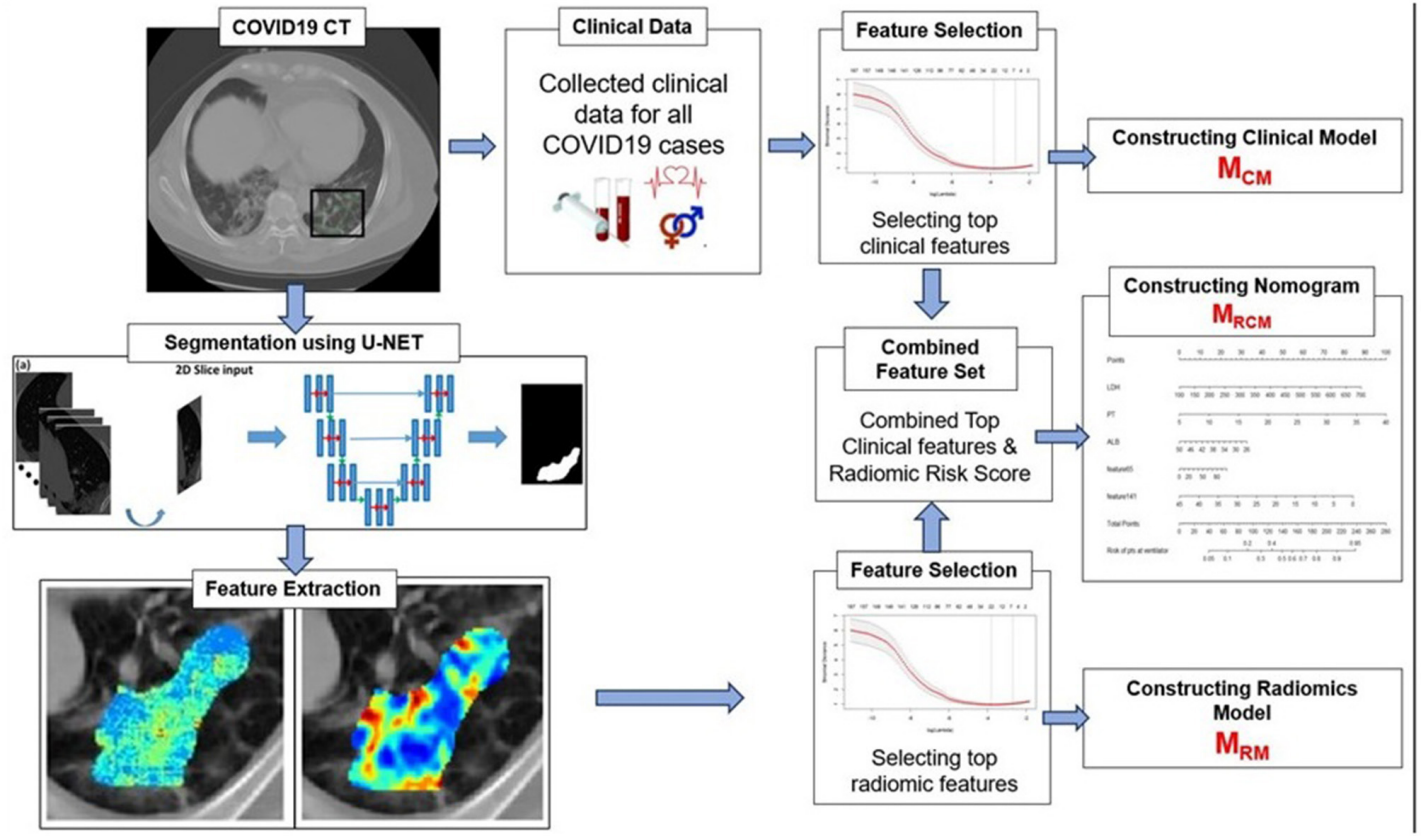
Workflow of the experiment. The first step involves segmentation of coronavirus disease (COVID) consolidations, which were further used for radiomic feature extractions. Next, the top clinical and radiomic features were selected using (LASSO) analysis and further used for constructing radiomic model (M_RM_), clinical model (M_CM_), and combined combined clinical–radiomic (MRCM) nomogram.

The primary endpoint of the study was predicting the severity of the COVID-19 disease, specifically, predicting patients who would require an invasive mechanical ventilator vs. those who would not. Figure 2 explains the entire experimental design pipeline.

First, to validate the automatic CNN-based segmentation model's performance, the Dice similarity coefficient (DSC) was used. The DSC was evaluated on the voxel-wise segmentation performance and compared against an expert radiologist reader.

For building the prediction models, the top features were selected from the entire feature pool using the LASSO algorithm on ${\text{D}}_{1}^{\text{T}}$ to constrict M_RM_ and M_CM_. LASSO provides a principled way to reduce the number of features in a model. LASSO penalizes the L1 norm of the weights, which induces sparsity in the solution (many weights are forced to zero). This performs variable selection (the “relevant” variables are allowed to have non-zero weights). The degree of sparsity is controlled by the penality term, which was selected within a 10-fold cross-validation setting. The M_RM_ model had top Radiomic features in the form of “radiomic score” constructed using the weighted sum of these features with their corresponding LASSO coefficients. The M_CM_ model consisted of top clinical features, and the final model, M_RCM_, was constructed using the top clinical features integrated with “radiomic score” in the form of nomogram analysis.

All three models were constructed with logistic regression (LR) classifiers. The receiver operating characteristic (ROC) and precision-recall (PR) analysis, along with sensitivity, specificity, and area under the curve (AUC), were used as performance metrics to evaluate the accuracy of the M_RM_, M_CM_, and M_RCM_. DeLong test was used to compare the statistical significance of differences between the models (34). Odds ratio (OR) and 95% confidence intervals (CI) were calculated to estimate the effect size of important clinical factors and image features. For ${\text{D}}_{1}^{\text{T}}$, cross-validation results were reported as mean ± standard deviation.

The final M_RCM_ model was represented as a clinico-radiomic nomogram (35). The patients were divided into high-risk (ventilator) groups and low-risk (non-ventilator) groups using the optimal cutoff point obtained from the LR model. The decision curve was plotted and evaluated to see the added improvement of the nomogram over the individual models. The net benefit was calculated by summing the benefits (true-positive results) and subtracting the harms (false-positive results), weighting the latter by a factor related to the relative harm of undetected disease severity with the harm of unnecessary ventilator treatment (36). In this analysis, the added improvement of the M_RCM_ model was shown over M_CM_ and M_RM_.

## Results

### Study Population and Characteristics

Table 1 lists the study population characteristics for the two institutions D_1_ and D_2_. The median age of the patients was 59 in D_1_ and 60 in D_2_. In D_1_ and D_2_, 41.9, 55.3% had a mild disease, whereas 58.1, 44.7% had a severe disease having ended up requiring invasive mechanical ventilation.

### Segmentation Model

**Figure 3:**
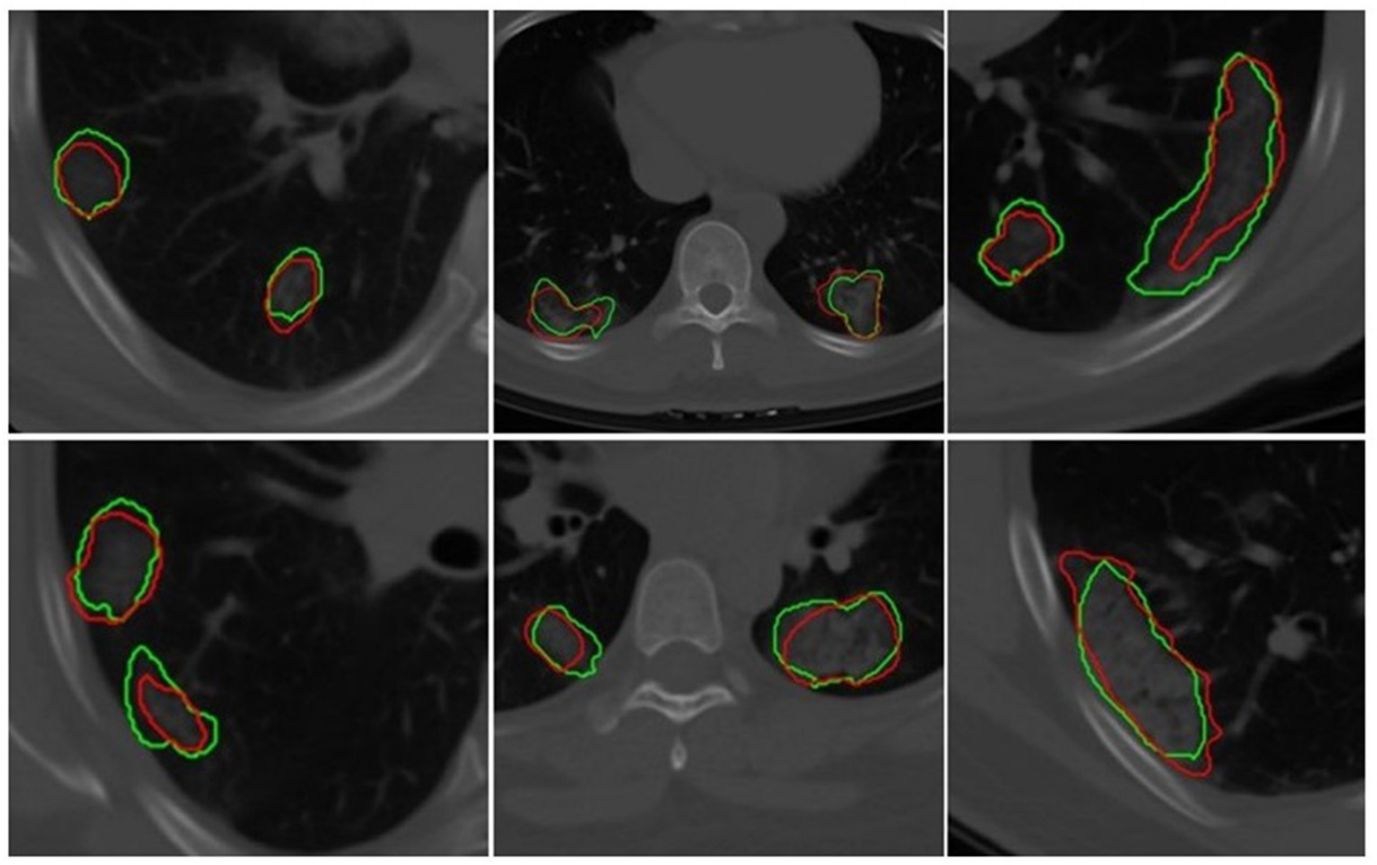
Segmentation results of a 2D U-Net in segmenting ground-glass opacities and COVID consolidation regions. Green contours represent ground-truth delineations of the ground-glass opacities (GGOs) and consolidations, and their corresponding network segmentation contours are represented in red. The 2D U-Net network was trained on the subset of the training set.

The U-Net network detected 1,017 of 1,260 COVID regions (3D connected components) annotated by the radiologist with 449 false positives on ${\text{D}}_{\text{UNET}}^{\text{T}}$. The corresponding sensitivity and positive predictive value (PPV) were found to be 80.71 and 69.3%, respectively. The output segmentation (Figure 3) by the network had an overlap of DSC = 0.60 ± 0.02 with ground-truth delineations for the detected regions. On the validation set of *N* = 96 (${\text{D}}_{\text{UNET}}^{\text{V}}$), 1,071 of 1,353 annotated regions were detected with 470 false positives, which resulted in a sensitivity of 79.15% and PPV of 69.5%. The corresponding DSC of the segmentation on ${\text{D}}_{\text{UNET}}^{\text{V}}$ was 0.59. The corresponding DSC of the segmentation on DVUNET was 0.59 (Table 2).

**Table 2:** U-Net-based model analysis.

	**Detected**	**False positives**	**Sensitivity (%)**	**PPV (%)**	**DSC**
${\text{D}}_{\text{UNET}}^{\text{T}}$	1,017/1,260	449	80.71%	69.3%	0.60 ± 0.02
${\text{D}}_{\text{UNET}}^{\text{V}}$	1,071/1,353	470	79.15%	69.5%	0.59

### Individual Radiomic- and Clinical-Based Machine Learning Models for Predicting Patients Being on the Ventilator for COVID-19 Patients

**Figure 4:**
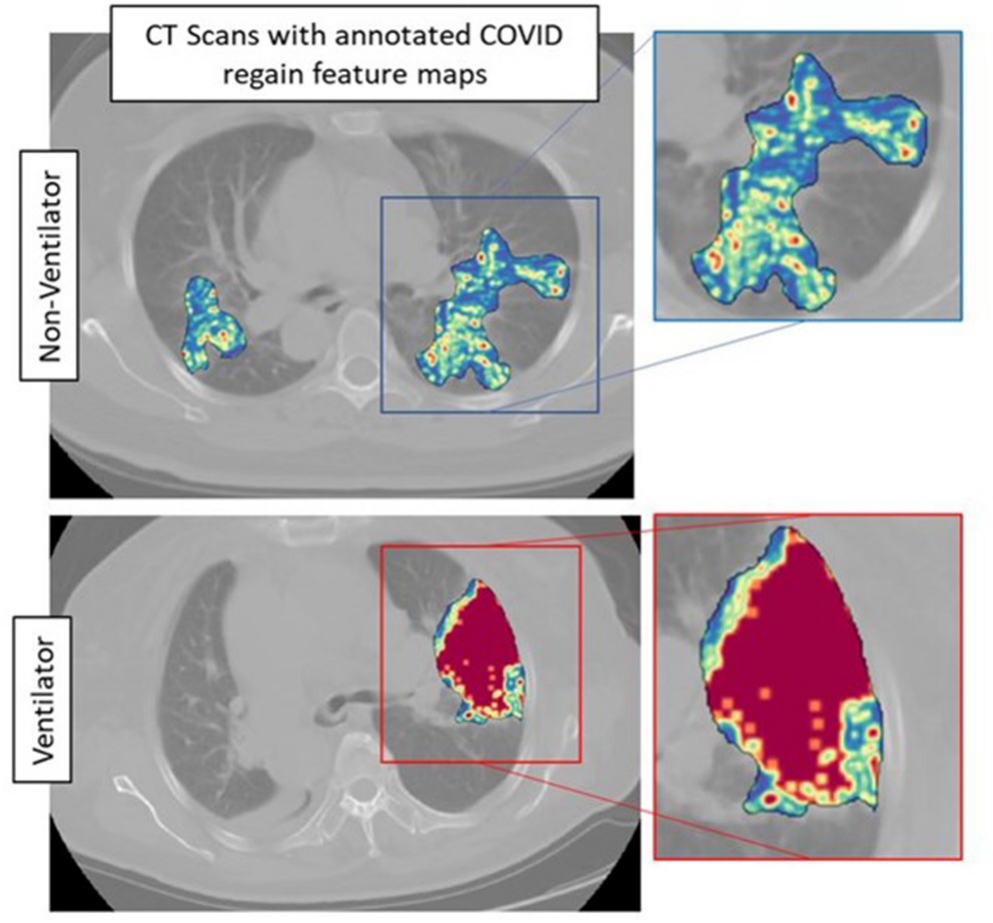
Feature maps of top selected features for ventilated (lower row) and non-ventilated (upper row) cases.

The top five features selected within the radiomic model using the LASSO analysis are listed in Table 3. Figure 4 shows the difference between feature maps for ventilator and non-ventilator cases. These features were statistically significant between the ventilator and non-ventilator groups, with higher feature values potentially representing patients at higher risk of disease. The violin plots of the top features are represented in Appendix 1.

**Table 3:** Selected top features.

**Feature family**		**Feature name**	**LASSO coefficient**
Texture features	GLCM	Inverse variance	1.65 e(0)
	GLRLM	High gray level run emphasis	1.96 e(−2)
	GLSZM	Small area low gray-level emphasis	8.95 e(−5)
First order		90th percentile pixel value	−3.48 e(−2)
Absolute infection size			2.38 e(−6)

The constructed logistic regression model with radiomic score (M_RM_) had an AUC of 0.754, 95% CI (0.709–0.799) on ${\text{D}}_{1}^{\text{T}}$. The same model gave an AUC of 0.836, 0.758, and 0.719 on ${\text{D}}_{1}^{\text{V}}$, ${\text{D}}_{2}^{\text{V}}$, and combined test set (${\text{D}}_{1}^{\text{V}}$ + ${\text{D}}_{2}^{\text{V}}$). For the clinical model, M_CM_, the LASSO method selected albumin (ALB), lactate dehydrogenase (LDH), and age as the most predictive parameters. Using the most discriminating clinical factors, the model trained yielded an AUC of 0.784, 95% CI: (0.743–0.825) on ${\text{D}}_{1}^{\text{T}}$ and 0.813, 0.688, and 0.703 on ${\text{D}}_{1}^{\text{V}}$, ${\text{D}}_{2}^{\text{V}}$, and combined test set (${\text{D}}_{1}^{\text{V}}$ + ${\text{D}}_{2}^{\text{V}}$), respectively.

Appendix 1 shows the violin plots for the top clinical features and scores for training and validation datasets.

### An Integrated Clinical and Imaging Nomogram to Predict the Need for Mechanical Ventilation in COVID-19 Patients

The integrated radiomic–clinical nomogram, M_RCM_, included the radiomic score and three clinical parameters—age, albumin, and lactate dehydrogenase. Table 4 shows the effect size and odds ratio for these variables.

The M_RCM_ model outperformed both M_CM_ and M_RM_, resulting in an AUC of 0.847 and 0.771, and 0.735 on ${\text{D}}_{1}^{\text{V}}$, ${\text{D}}_{2}^{\text{V}}$, and combined ${\text{D}}_{1}^{\text{V}}$ + ${\text{D}}_{2}^{\text{V}}$ test set, respectively.

**Figure 5:**
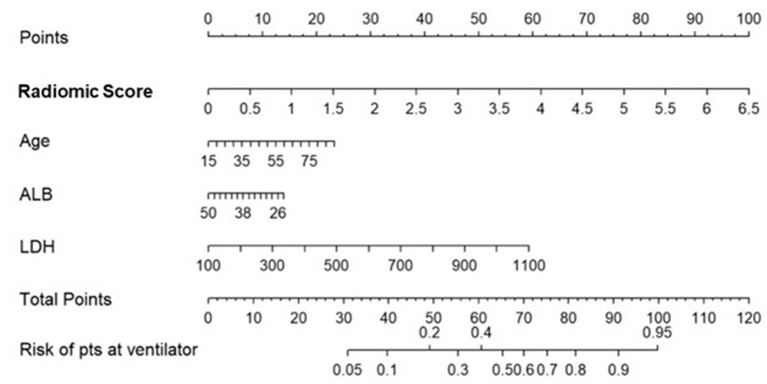
Constructed nomogram, M_RCM_, which included radiomic score, age, albumin (ALB), and lactate dehydrogenase (LDH). The nomogram calculates the probability of the patient being on the ventilator.

The multivariate logistic regression analysis of the M_RCM_ nomogram showed that the radiomic score was found to add independent prognostic value to the M_RCM_ model. The predicted score of 0.54 or greater [an optimal cutoff point on the receiver operating characteristic (ROC) curve] suggested the need for mechanical ventilation, while scores ≤ 0.54 could be managed conservatively (Figure 5). Additionally, the AUC comparison within the three models showed that the increase in AUC in M_RCM_ was statistically significant when compared against the clinical model M_CM_.

**Figure 6:**
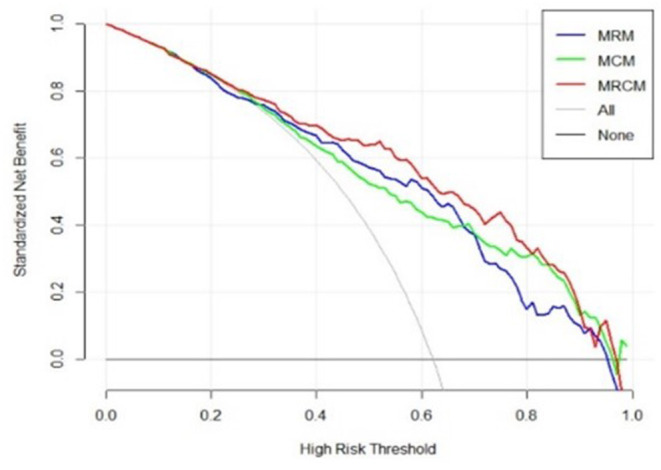
Decision curve analysis of M_RCM_ (clinical and imaging integrated nomogram) constructed using developed radiomic score, age, and three laboratory parameters (LDH and ALB). The other (bottom) were clinical (M_CM_) and radiomic model (M_RM_).

The decision curve analysis indicated an added net benefit using the integrated model M_RCM_ over M_CM_ and M_RM_ (Figure 6). The combined M_RCM_ model had the highest net benefit compared with M_CM_, M_RM_, and simple strategies, such as treating all patients (light vertical curve line) or treating no patients (horizontal black line) across the full range of threshold probabilities.

## Discussion

In this study, we presented an integrated radiomic and clinical nomogram (M_RCM_) to predict at baseline patients with a severe phenotype of COVID-19 and who would end up needing mechanical ventilation and intubation. We explicitly used patients with baseline CT scans and laboratory parameters observed within the milder stage of the disease to reduce the bias. M_RCM_ comprised a radiomic score constructed using the annotated GGO and consolidation regions on lung CT scans along with age, albumin (ALB), and lactate dehydrogenase (LDH). Meanwhile, the radiomic model (M_RM_) incorporated the radiomic score constructed using five radiomic features. The clinical model (M_CM_) was built using age, albumin, and lactate dehydrogenase out of routine clinical laboratory parameters. We constructed a U-NET-based segmentation algorithm to segment COVID-19 regions from the baseline CT scans to completely automate the whole process. The three models were trained and independently validated on a large multi-institutional dataset making this the most extensive study to date involving AI and radiomics for the prognosis of COVID-19 patients.

Our radiomic model, M_RM_, incorporated radiomic score constructed using top features observed from within the gray-level matrix-based feature families explaining textural patterns of COVID regions. These features had higher expression in potentially high-risk cases, suggesting a more chaotic and disturbed microarchitecture in patients at a higher risk of disease (Figure 4). Our results are in line with results presented by Wu et al. (14), where four features out of five were observed from gray-level matrix-based feature family. The higher textural value from the gray-level co-occurrence matrix indicates the more abnormal lung tissues, which further seemed to be associated with the worse outcome. This is consistent with previous findings that show that peripheral, diffuse distributions and paving patterns are associated with poor survival in COVID-19 cases (37). Compared with the usual imaging CT model features, radiomics offer superior performance in the COVID-19 space. Simply looking at radiomic models for predicting the severity of COVID-19 patients, the signatures constructed using SVM by Fu et al. (24) achieved an AUC of 0.83 on *N* = 64, and Wei (25) achieved an AUC of 0.93 on *N* = 81. Our results show a better performance considering that we had larger datasets with completely independent multi-institutional validation sets.

The most prognostic clinical variables observed within the clinical model were age, ALB, and LDH selected using the LASSO. A low level of ALB was associated with poorer outcomes, i.e., the patient being on the ventilator (32). In contrast, low levels of LDH were associated with better outcomes (32, 33). The boxplots of these features are depicted in Appendix 1. ALB and LDH are considered biomarkers for predicting the COVID-19 severity in the previously published findings (32). We observed the third important clinical feature to be the patient's age, where an advanced age was associated with a worse outcome for COVID19 patients (38).

The integrated M_RCM_ model outperformed M_RM_ and M_CM_ models in predicting which COVID-19 patients would ultimately need invasive mechanical ventilation on both internal and external validation sets ${\text{D}}_{1}^{\text{V}}$ and ${\text{D}}_{2}^{\text{V}}$. M_RCM_ improved performance by over ~2.5% over M_RM_ and ~3.77% over M_CM_ in terms of AUC, with the performance increase statistically significant by DeLong's test. The M_RCM_ model was used to individualize risk assessments. The predicted score of 0.54 or greater [an optimal cutoff point on the receiver operating characteristic (ROC) curve that had an optimal balance between sensitivity and specificity] suggested the need for mechanical ventilation, while scores ≤ 0.54 could be managed conservatively. We only noticed one nomogram approach developed by Yu et al. (39), which used age, density, perfusion signs, and severity score of lungs constructed by assessing each lobe of the lung for predicting the severity of COVID-19. The nomogram achieved an AUC of 0.929 (95% CI, 0.889–0.969) on training (*N* = 152) and 0.936 (95% CI, 0.867–1.000) on the validation set (*N* = 65), but their analysis did not involve radiomics. Our developed nomogram was completely automated, had minimal involvement of a radiologist, and achieved almost comparable results within larger datasets.

The previous work on combining radiomics with clinical variables shows promising results for predicting disease severity. For the combined clinical and radiomic model, in the work by Chao et al. (13), the authors integrated the L/W ratio, lymphocyte count, WBC, and age into whole lung radiomics to achieve the highest AUC of 0.88 in predicting the need for ICU admission. The advantage in our approach compared with previous ones includes a higher number of cases and a nomogram representation.

In the recent study by Roberts et al. (40), the authors point out that many recent AI/machine learning studies on diagnosis and prognosis of COVID-19 from radiographic scans are not reproducible and would not be clinically deployable. Furthermore, they point out that many studies within this space have not been stress tested or validated on independent external test sets. Many of these models have not assessed model sensitivity or robustness and have methodological flaws and/or underlying biases. In our work, we have attempted to deliberately and purposefully develop, validate, and analyze our approach in a more rigorous manner, including validating this model on one of the largest external test sets reported to date.

Despite the favorable prognostic efficacy of the clinico-radiomic nomogram, we acknowledge that our approach does have its limitations. First, our study was retrospective, and the two cohorts were not homogeneously defined. To ensure the clinical usefulness of M_RCM_, we need to validate the tool in a prospective setting by following up with patients until discharge. Second, the study's retrospective nature also precluded us from standardizing the time between RT-PCR and CT scans across the cohort. Finally, we did not explicitly compare segmentation and prediction performances between the AI model and expert radiologist interpretations. We will attempt to address these limitations in future work.

## Conclusion

We presented an integrated radiomic and clinical parameter-based prognostic model using routinely available blood parameters and standard-of-care CT scans at baseline in SARS-CoV2-positive patients at the milder stage of the disease. We showed in a multi-institutional cohort that our integrated model had a good performance in identifying which of these patients would decline in severe respiratory distress with need for intubation and mechanical ventilation. Further multisite prospective validation would allow for the clinical deployment of M_RCM_, especially to triage patients for ventilator usage, in the face of worldwide shortages in the availability of mechanical ventilators. The developed tool, once prospectively validated, could provide an objective way to risk stratifying patients immediately following diagnosis with COVID-19.

## Data Availability Statement

The original contributions presented in the study are included in the article/Supplementary Materials, further inquiries can be directed to the corresponding author.

## Ethics Statement

The Institutional Review Board Committee approved the retrospective chart review study of record at the University Hospitals, Cleveland (STUDY20200463), and the Renmin Hospital of Wuhan University (ethics number: V1.0; IRB number 2020KS02010). The need for written consent was waived.

## Author Contributions

PV, KB, AH, MA, and AM were involved in the study design. PV and MA performed the radiomics analysis. AH performed DL analysis. PF helped with the statistical analysis. AG, JF, KA, RG, LY, CL, and MJ collected the data. PV wrote the initial draft. AM was responsible for the decision to submit the manuscript. All authors reviewed, contributed and approved the manuscript, and had access to all the data.

## Funding

Research reported in this publication was supported by the National Cancer Institute under award numbers 1U24CA199374-01, R01CA249992-01A1, R01CA202752-01A1, R01CA208236-01A1, R01CA216579-01A1, R01CA220581-01A1, R01CA257612-01A1, 1U01CA239055-01, 1U01CA248226-01, 1U54CA254566-01, National Heart, Lung and Blood Institute 1R01HL15127701A1, R01HL15807101A1, National Institute of Biomedical Imaging and Bioengineering 1R43EB028736-01, National Center for Research Resources under award number 1 C06 RR12463-01, VA Merit Review Award IBX004121A from the United States Department of Veterans Affairs Biomedical Laboratory Research and Development Service the Office of the Assistant Secretary of Defense for Health Affairs, through the Breast Cancer Research Program (W81XWH-19-1-0668), the Prostate Cancer Research Program (W81XWH-15-1-0558, W81XWH-20-1-0851), the Lung Cancer Research Program (W81XWH-18-1-0440, W81XWH-20-1-0595), the Peer Reviewed Cancer Research Program (W81XWH-18-1-0404), the Kidney Precision Medicine Project (KPMP) Glue Grant, the Ohio Third Frontier Technology Validation Fund, the Clinical and Translational Science Collaborative of Cleveland (UL1TR0002548) from the National Center for Advancing Translational Sciences (NCATS) component of the National Institutes of Health and NIH roadmap for Medical Research. The Wallace H. Coulter Foundation Program in the Department of Biomedical Engineering at Case Western Reserve University. Sponsored research agreements from Bristol Myers-Squibb, Boehringer-Ingelheim, and Astrazeneca. The authors declare that this study received funding from Bristol Myers-Squibb, Boehring-Ingelheim, and AstraZeneca. The funders were not involved in the study design, collection, analysis, interpretation of data, the writing of this article, or the decision to submit it for publication.

## Author Disclaimer

The content is solely the responsibility of the authors and does not necessarily represent the official views of the National Institutes of Health, the US Department of Veterans Affairs, the Department of Defence, or the United States Government.

## Conflict of Interest

AM reports grants from the National Cancer Institute of the National Institutes of Health, grants from the National Center for Research Resources, grants from the VA Merit Review Award, grants from the DOD Lung Cancer Investigator-Initiated Translational Research Award, during the conduct of the study, grants from the DOD Prostate Cancer Idea Development Award, grants from the DOD Peer Reviewed Cancer Research Program, grants from the National Institute of Diabetes and Digestive and Kidney Diseases, grants from the Ohio Third Frontier Technology Validation Fund, grants from the Wallace H. Coulter Foundation Program in the Department of Biomedical Engineering and the Clinical and Translational Science Award Program (CTSA) at Case Western Reserve University, grants from the Department of Defense Peer Reviewed Cancer Research Program (PRCRP) Career Development Award, grants from the Dana Foundation David Mahoney Neuroimaging Program. The remaining authors declare that the research was conducted in the absence of any commercial or financial relationships that could be construed as a potential conflict of interest.

## Publisher's Note

All claims expressed in this article are solely those of the authors and do not necessarily represent those of their affiliated organizations, or those of the publisher, the editors and the reviewers. Any product that may be evaluated in this article, or claim that may be made by its manufacturer, is not guaranteed or endorsed by the publisher.

## Abbreviations

COVID-19: the coronavirus disease 2019
SARS-Cov-2: respiratory syndrome coronavirus 2
ARDS: acute respiratory distress syndrome
CT: computed tomography
RT-PCR: reverse transcription polymerase chain reaction
AI: artificial intelligence
CNN: convolutional neural network
DL: deep learning
GGO: ground glass opacities
DSC: Dice similarity coefficient
ROC: receiver operating characteristic curve
PR: precision recall
AUC: area under the receiver operating characteristic curve
LDH: lactate dehydrogenase
ALB: albumin
LASSO: least absolute shrinkage and selection operator
GLCM: gray-level co-occurrence matrix
GLSZM: gray-level size zone matrix
GLRLM: gray-level run length matrix
NGTDM: neighboring gray tone difference matrix
GLDM: gray-level dependence matrix
M_RM_: radiomic-based model
M_CM_: clinical-based model
M_RCM_: radiomic–clinical-based nomogram.
