# Supplementary material for: An End-to-End Integrated Clinical and CT-Based Radiomics Nomogram for Predicting Disease Severity and Need for Ventilator Support in COVID-19 Patients: A Large Multisite Retrospective Study
Source: Front Radiol. 2022 Apr 8;2:781536. doi: 10.3389/fradi.2022.781536 (PMC9696643; doi:10.3389/fradi.2022.781536)
Supplement: Supplementary file 1 [file Data_Sheet_1.PDF]

Appendix-1

Section 1:

Table S.1 – Studies looking at Radiomics in COVID-19 from Dec 2019 to Dec 2020

	Title	Type; #of patients	Experiments Performed	Are clinical factors included?	Final Model Performance
1.	Hypergraph learning for identification of COVID-19 with CT imaging[1]	Diagnostic (Identifying COVID-19). N=3330	Used hypergraph learning on regional features and radiomic features	---	Accuracy 0.898 (+/-0.2)
2.	Radiomics nomogram for the prediction of 2019 novel coronavirus pneumonia caused by SARS-CoV-2[2]	Diagnostic (Identifying COVID-19). N=329	Constructed Radiomics Score and integrated with clinical parameters to construct a combined nomogram	Distribution, maximum lesion, hilar, and mediastinal lymph node enlargement; pleural effusion	Train AUC: 0.959 (95% CI 0.9333-0.985) Test AUC: 0.955 (95% CI 0.899-0.995)
3.	A Quantitative and Radiomics approach to monitoring ARDS in COVID-19 patients based on chest CT: a retrospective cohort study[3]	Diagnostic (Identifying ARDS from CT images) N=86	Three models were constructed using quantitative imaging, radiomic features and combination	Age, total volume, area proportion of zone 10	Train AUC: 0.97 (95% CI 0.94–0.99) Test AUC: 0.94 (95% CI 0.88–0.99)
4.	The study of automatic machine learning base on radiomics of non-focus area in the first chest CT of different clinical types of COVID-19 pneumonia[4]	Diagnostic (predicting COVID-19 & its type by analyzing the non-focus area of the lung); N=216	Focused Radiomics Analysis to create three models (moderate Vs. severe, Moderate Vs. Control and Severe Vs. Control)	---	Distinguishing Moderate to Severe COVID19 - Train AUC 0.98 Test AUC 0.95
5.	Radiomics-based model for accurately distinguishing between severe acute respiratory syndrome associated coronavirus 2 (SARS-CoV2) and influenza A infected pneumonia[5]	Diagnostic (identifying COVID-19 from IAP); N=177	Constructed Radiomic Score for predicting COVID-19	---	AUC: 0.87 (95% CI: 0.77-0.93)
6.	Decoding COVID-19 pneumonia: comparison of	Diagnostic (identifying	Combined extracted Deep Learning and Radiomics		AUC 0.98

	deep learning and radiomics CT image signatures[6]	COVID-19 pneumonia); N=266	features to predict COVID-19.		
7.	Discrimination of pulmonary ground-glass opacity changes in COVID-19 and non-COVID-19 patients using CT radiomics analysis[7]	Diagnostic (Identifying COVID-19); N=301	Constructed Model using Radiomics	---	AUC 0.905
8.	Predicting Mechanical Ventilation Requirement & Mortality in COVID-19 using Radiomics and Deep Learning on Chest Radiographs: A Multi-Institutional Study[8]	Prognostic (Predicting mechanical ventilation & mortality); N = 514	Embedded CNN based model and Radiomic based classifier to predict the outcome	---	Ventilator requirement Test AUC 0.905 Mortality Test AUC 0.926
9.	Integrative Analysis for COVID-19 Patient Outcome Prediction[9]	Prognostic (need for intensive care unit (ICU) admission); N=295	Integrated whole lung Radiomics, non-imaging clinical, laboratory and demographic data	L/W ratio; Lym count; WBC; Ag	0.884 (95% CI: 0.875, 0.893) on Data-Site1
10	Machine learning-based CT radiomics method for predicting hospital stay in patients with pneumonia associated with SARS-CoV2 infection: a multicenter study[10]	Prognostic (Predicting long or short-term hospital stay of COVID-19 patients); N=31 (with 76 lesions)	CT radiomics model with RF and LR machine leaning classifiers.		Test AUC= 0.97 (95% CI, 0.83–1.0)
11	Identification of common and severe COVID-19: the value of CT texture analysis and correlation with clinical characteristics[11]	Prognostic (Differentiate severe from common COVID-19 patients); N=81	Constructed radiomics model and clinical model using LR. Compared clinical features with Radiomic Textural features using spearman correlation.		AUC 0.93 (95% CI ,0.86–1.00)
12	A Novel Machine Learning derived Radiomic Signature	Prognostic (Differentiate	Radiomic signature constructed using SVM.		AUC 0.8333

	of the Whole Lung Differentiates Stable From Progressive COVID-19 Infection: A Retrospective Cohort Study[12]	stable and progressive COVID-19); N=64	The model evaluated with various subset analysis, and correlated with clinical, laboratory and CT findings		
13	CT Quantification and Machine-learning Models for Assessment of Disease Severity and Prognosis of COVID-19 Patients[13]	Prognosis (Assessment of disease severity); N=99	Created two models to distinguish severe + critical patients using Radiomics, Clinical, and hybrid model		Moderate vs. (severe + critical) 0.927(95% CI, 0.922-0.931) Severe vs. critical 0.929 (95% CI , 0.924-0.934)
14	Radiomics Analysis of Computed Tomography helps predict poor prognostic outcome in COVID-19[14]	Prognosis (Predicting survival to death, need for mechanical ventilation, or intensive care unit admission); N=492	Constructed CrrScore for predicting overall and 28-day outcome. Integrated with clinical parameters. Compared laboratory parameters with CrrScore.	Age; Comorbidity and type	C-Index=0.850
15	CT Radiomics, Radiologists and Clinical Information in Predicting Outcome of Patients with COVID-19 Pneumonia[15]	Prognostic (Predicting disease outcome and severity of COVID19 patients); N=315	Constructed Radiomics, clinical and integrated models to predict the disease outcome and severity	SpO2; Astma; altered mental status	Predicting patient outcome AUC: 0.85
16	A model based on CT radiomic features for predicting RT-PCR becoming negative in coronavirus disease 2019	Treatment Response (Predicting RT-PCR	Clinical characteristics, Radiomic and quantitative Features were selected to construct logistic	Time interval from symptoms onset to CT	Training - 0.811 and testing - 0.812

	(COVID-19) patient[16]	negativity during clinical treatment); N=20	regression model	exams	

Section 2: Data-Preprocessing

Before extracting the Radiomic features, all the CT scans were resampled to the size of [0.75, 0.75, 5] using linear interpolation. All the cases were resampled to this specific dimension because 745 cases out of a total of 897 cases had 0.75 by 0.75 by 5 dimensions. The radiomic features were extracted from the resampled scans using MATLAB-2015 and Center of Computational Imaging and Personalized Diagnostics (CCIPD) inbuilt feature extraction pipeline. The features from the training and testing cohorts were standardized with respect to training dataset before using within the machine learning models.

Section 3: Programming Languages

Most of the analysis in this study was done using MATLAB2015 and "R" for statistical analysis. LASSO feature selection model was used using the ’glmnet’ library in R. The nomogram was constructed using the package “rms”. Area under the receiver characteristic operating curve (AUC) was calculated using R package ’pROC’. A trapezoidal rule was used along with 95% confidence interval (CI) obtained by performing 2000 stratified bootstrap replicates to calculate AUCs. A DeLong test was used to compare the difference between two AUCs. The boxplots were plotted using package ’ggplot2’.

Section 4: CT Parameters

Table S.2 – CT parameters for D-1 Renmin Hospital, Wuhan

**Criteria**			**Reconstruction Kernels Used**
**Vendor**	GE medical systems (GE)	745	‘BONEPLUS’, ‘LUNG’, ‘CHEST’, ‘STANDARD’
	United Imaging Healthcare (UIH)	42	‘B_SHARP_C’, ‘B_SOFT_B’
**Slice Thickness**	5 mm	787	

Table S.3 – CT parameters for D-2 University Hospitals, US

**Criteria**			**Reconstruction Kernels Used**
**Vendor**	GE medical systems (GE)	4	‘STANDARD’
	Phillips	34	‘B’, ‘C’, ‘YA’, ‘YB’
	Siemens	9	‘B30f’, ‘B50f’
**Slice Thickness**	2 mm	24	
	3 mm	16	

	5 mm	7	

Section 5: U-Net based segmentation model

For segmenting the COVID related consolidations, we used a U-Net based model as described in the following diagram. The diagram represents details regarding U-Net architecture.

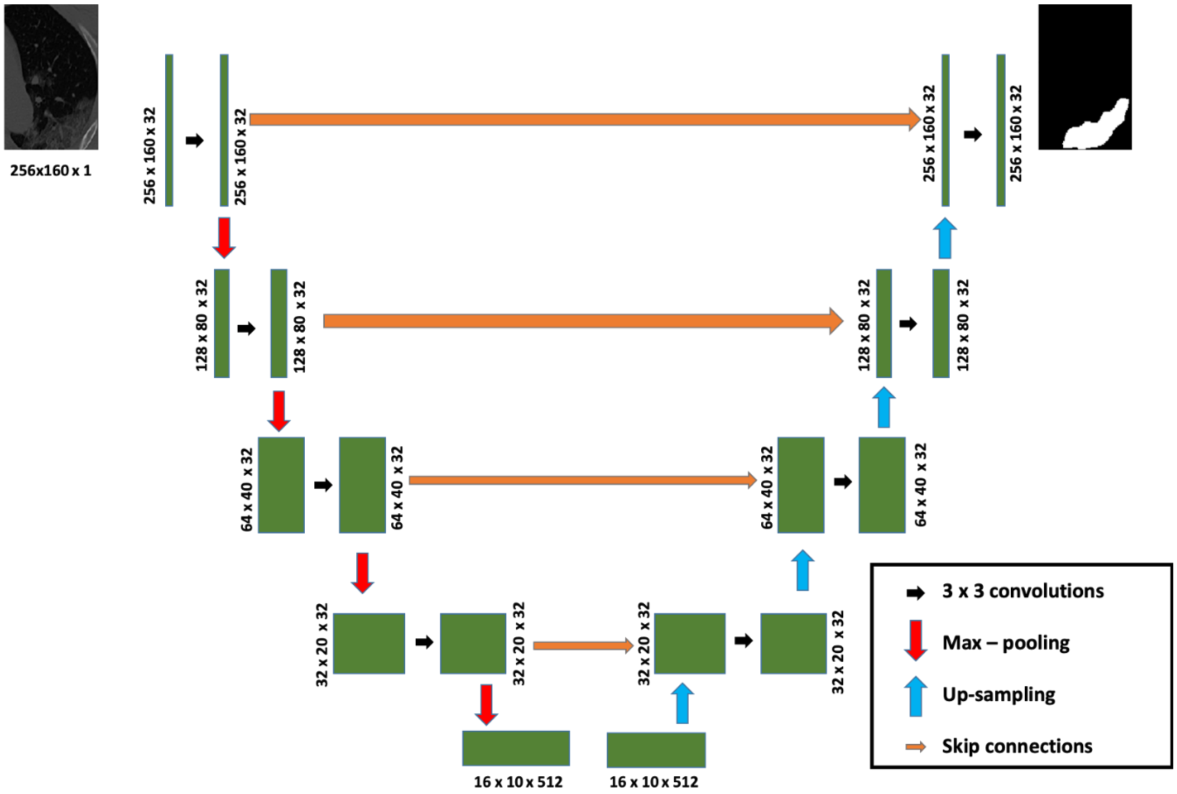

Figure 1- Architectural diagram of a 2D U-Net used for segmentation of ground-glass opacities and consolidations. U-Net consists of an encoder block and a decoder block. Both encoder and decoder have 5 convolutional blocks with two convolutional layers in each block.

Section 6: Total Extracted Radiomic Features.

Table S.4 – Radiomic Features extracted from the annotated COVID consolidations

Family	Feature Family Description
**Infection Size**	
**GLCM**	If I is an image with N discrete grey levels, the GLCM matrix is constructed by looking at the combination of neighboring pixels.<br>The (i,j)th element of this matrix represents the number of times the combination of levels ii and jj occur in two pixels in the image separated by a distance of x pixels along angle θ.
**Grey Level Size Zone Matrix**	It quantifies gray level zones in an image. A gray-level zone is defined as a number of

	connected voxels that share the same gray level intensity. In a gray level size zone matrix (i,j)th(i,j)th element equals the number of zones with gray level I and size j appear in an image.
**Gray level run length matrix**	It quantifies gray level runs, defined as the number of pixels' length, of consecutive pixels with the same gray level value. In a gray level run length matrix, the (i,j)th element describes the number of runs with gray level i and length j occur in the image (ROI) along angle θ
**Neighboring Gray Tone Difference Matrix**	It quantifies the difference between a gray value and its neighbors' average gray value within distance x. The sum of absolute differences for gray level i is stored in the matrix.
**Gray level dependence matrix**	It quantifies gray level dependencies in an image. A gray level dependency is defined as the number of connected voxels within distance x that are dependent on the center voxel. A neighboring voxel with gray level j is considered dependent on the center voxel with gray level i if $\|i-j\| \leq \alpha$. In a gray level dependence matrix, the $(i,j)^{th}$ element describes the number of times a voxel with gray level ii with jj dependent voxels in its neighborhood appears in the image.

Section 7: Clinical Features.

Table S.5 – Clinical Features used for the analysis and differences between Dataset-1 and Dataset-2

Variable	D1 (N = 787)	D2 (N = 110)	Statistics	P-Value
Age	59 [18-96]	62 [20-94]	-2.45	0.014
Urea	4.71 [1.84-51.34]	1.9 [0.4-21.5]	11.20	<0.001
ALB	38.2 [22.7-50.6]	36 [25-44]	4.07	<0.001
LDH	229 [108-1039]	348.5 [127-919]	-5.73	<0.001
AST	24 [10-2104]	30 [10-419]	-3.12	0.002
ALT	23 [3-1630]	21 [5-282]	0.879	**0.378**
WCC	5.57 [0-97]	6.05 [2.2-33.9]	-1.926	**0.054**
LYM	1.21 [0.1-15.28]	1.05 [0.23-3.47]	2.545	0.011
Neu	3.6 [0.2-32.94]	4.6 [0.59-31.2]	-3.347	0.008
Mono	0.47 [0.04-5.61]	0.45 [0.0 -1.76]	1.39	**0.163**
PT	117 [94-385]	134 [100-329]	-9.32	<0.001
Cr	0.6 [0.3-13.8]	1.13 [0.4-10.96]	-11.4	<0.001

Section 7: Box-Plots of variables used in nomogram for train and test datasets.

The following figure shows the violin plots for top clinical features and radiomic score for training as well as validation datasets.

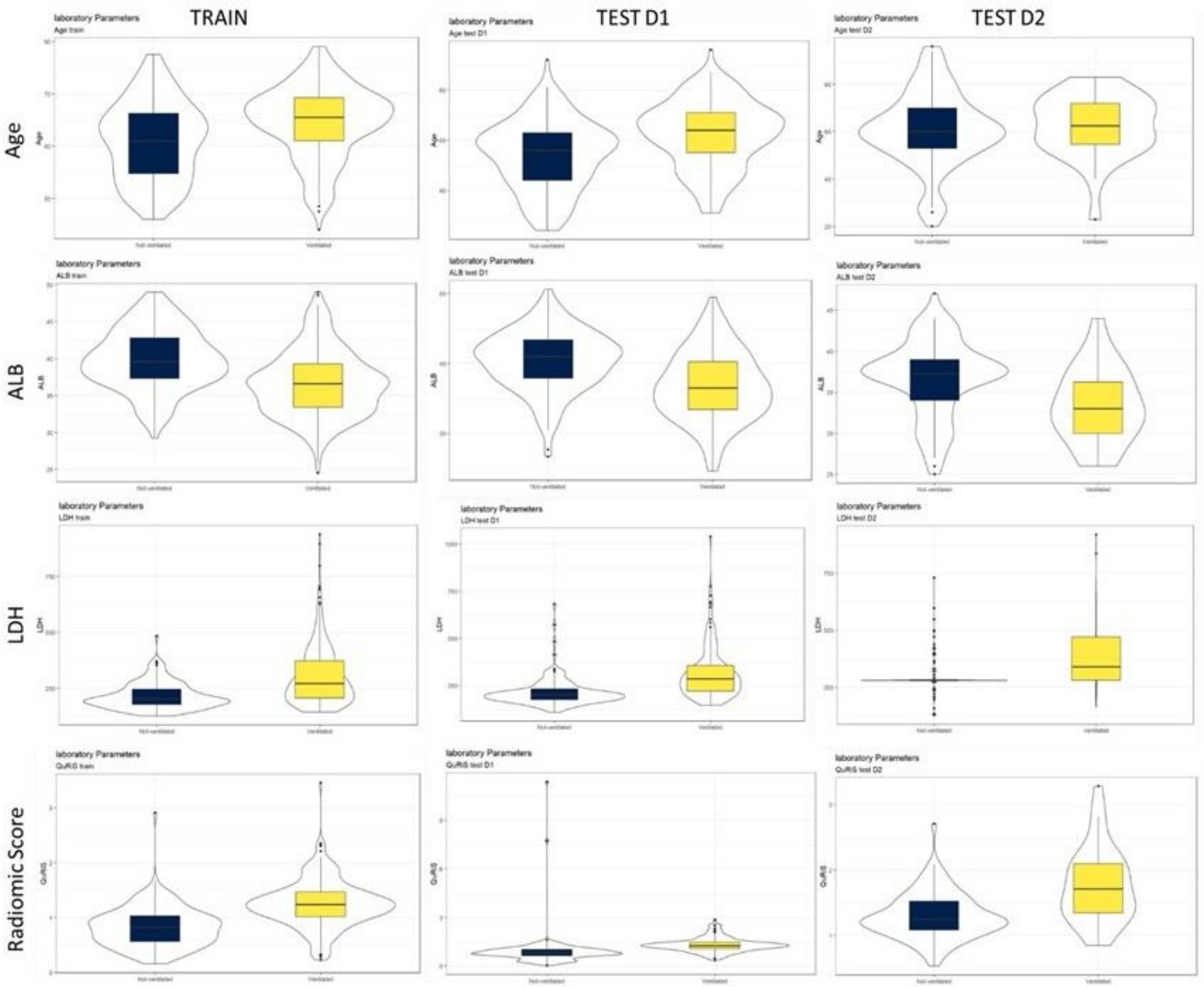

Figure 2-Violin-Plots of top clinical features and Radiomic Score for training

Section 8: Matthew's coefficients, PPV, NPV values of the classifiers

Table S.5 –Matthews's coefficient, PPV, and NPV for all the training and validation datasets

		Matthews Coeff.	PPV	NPV
D_1^T	M_{RM}	0.399	0.854	0.653
	M_{CM}	0.432	0.865	0.548
	M_{RCM}	0.479	0.888	0.570
D_1^V	M_{RM}	0.531	0.814	0.714
	M_{CM}	0.464	0.823	0.634
	M_{RCM}	0.557	0.877	0.671

**D_2^V**	M_{RM}	0.426	0.606	0.816
	M_{CM}	0.415	0.582	0.837
	M_{RCM}	0.452	0.654	0.793
$D_1^V + D_2^V$	M_{RM}	0.437	0.807	0.634
	M_{CM}	0.419	0.794	0.627
	M_{RCM}	0.461	0.768	0.692

References:

1. Di D, Shi F, Yan F, Xia L, Mo Z, Ding Z, Shan F, Song B, Li S, Wei Y, Shao Y, Han M, Gao Y, Sui H, Gao Y, Shen D. Hypergraph learning for identification of COVID-19 with CT imaging. *Med Image Anal*. 2021 Feb 1;**68**:101910. doi:10.1016/j.media.2020.101910

2. Fang X, Li X, Bian Y, Ji X, Lu J. Radiomics nomogram for the prediction of 2019 novel coronavirus pneumonia caused by SARS-CoV-2. *Eur Radiol*. 2020 Jul 3;1–14. doi:10.1007/s00330-020-07032-z PMID: 32621237 PMCID: PMC7332742

3. Chen Y, Wang Y, Zhang Y, Zhang N, Zhao S, Zeng H, Deng W, Huang Z, Liu S, Song B. A Quantitative and Radiomics approach to monitoring ARDS in COVID-19 patients based on chest CT: a retrospective cohort study. *Int J Med Sci*. 2020 Jul 6;**17**(12):1773–1782. doi:10.7150/ijms.48432 PMID: 32714080 PMCID: PMC7378656

4. Tan H-B, Xiong F, Jiang Y-L, Huang W-C, Wang Y, Li H-H, You T, Fu T-T, Lu R, Peng B-W. The study of automatic machine learning base on radiomics of non-focus area in the first chest CT of different clinical types of COVID-19 pneumonia. *Sci Rep*. 2020 Nov 3;**10**. doi:10.1038/s41598-020-76141-y PMID: 33144676 PMCID: PMC7641115

5. Zeng Q, Zheng KI, Chen J, Jiang Z, Tian T, Wang X, Ma H, Pan K, Yang Y, Chen Y, Zheng M. Radiomics-based model for accurately distinguishing between severe acute respiratory syndrome associated coronavirus 2 (SARS-CoV-2) and influenza A infected pneumonia. *Medcomm*. 2020 Aug 13; doi:10.1002/mco2.14 PMID: 32838396 PMCID: PMC7436469

6. Wang H, Wang L, Lee EH, Zheng J, Zhang W, Halabi S, Liu C, Deng K, Song J, Yeom KW. Decoding COVID-19 pneumonia: comparison of deep learning and radiomics CT image signatures. *Eur J Nucl Med Mol Imaging*. 2020 Oct 23;1–9. doi:10.1007/s00259-020-05075-4 PMID: 33094432 PMCID: PMC7581467

7. Xie C, Ng M-Y, Ding J, Leung ST, Lo CSY, Wong HYF, Vardhanabhuti V. Discrimination of pulmonary ground-glass opacity changes in COVID-19 and non-COVID-19 patients using CT radiomics analysis. *Eur J Radiol Open*. 2020;**7**:100271. doi:10.1016/j.ejro.2020.100271 PMID: 32959017 PMCID: PMC7494331

8. Bae J, Kapse S, Singh G, Phatak T, Green J, Madan N, Prasanna P. Predicting Mechanical Ventilation Requirement and Mortality in COVID-19 using Radiomics and Deep Learning on Chest Radiographs: A Multi-Institutional Study. *ArXiv200708028 Cs Eess Q-Bio*. 2020 Jul 15;

9. Chao H, Fang X, Zhang J, Homayounieh F, Arru CD, Digumarthy SR, Babaei R, Mobin HK, Mohseni I, Saba L, Carriero A, Falaschi Z, Pasche A, Wang G, Kalra MK, Yan P. Integrative analysis for COVID-19 patient outcome prediction. *Med Image Anal*. 2021 Jan 1;**67**:101844. doi:10.1016/j.media.2020.101844

10. Yue H, Yu Q, Liu C, Huang Y, Jiang Z, Shao C, Zhang H, Ma B, Wang Y, Xie G, Zhang H, Li X, Kang N, Meng X, Huang S, Xu D, Lei J, Huang H, Yang J, Ji J, Pan H, Zou S, Ju S, Qi X. Machine learning-based CT radiomics method for predicting hospital stay in patients with pneumonia associated with SARS-CoV-2 infection: a multicenter study. *Ann Transl Med*. 2020 Jul;**8**(14). doi:10.21037/atm-20-3026 PMID: 32793703 PMCID: PMC7396749

11. Identification of common and severe COVID-19: the value of CT texture analysis and correlation with clinical characteristics | SpringerLink [Internet]. [cited 2020 Dec 12]. Available from: https://link.springer.com/article/10.1007/s00330-020-07012-3

12. Fu L, Li Y, Cheng A, Pang P, Shu Z. A Novel Machine Learning-derived Radiomic Signature of the Whole Lung Differentiates Stable From Progressive COVID-19 Infection. *J Thorac Imaging*. 2020 Nov;**35**(6):361–368. doi:10.1097/RTI.0000000000000544 PMID: 32555006 PMCID: PMC7682797

13. Cai W, Liu T, Xue X, Luo G, Wang X, Shen Y, Fang Q, Sheng J, Chen F, Liang T. CT Quantification and Machine-learning Models for Assessment of Disease Severity and Prognosis of COVID-19 Patients. *Acad Radiol*. 2020 Dec;**27**(12):1665–1678. doi:10.1016/j.acra.2020.09.004 PMID: 33046370 PMCID: PMC7505599

14. Wu Q, Wang S, Li L, Wu Q, Qian W, Hu Y, Li L, Zhou X, Ma H, Li H, Wang M, Qiu X, Zha Y, Tian J. Radiomics Analysis of Computed Tomography helps predict poor prognostic outcome in COVID-19. *Theranostics*. 2020 Jun 5;**10**(16):7231–7244. doi:10.7150/thno.46428 PMID: 32641989 PMCID: PMC7330838

15. Homayounieh F, Ebrahimian S, Babaei R, Karimi Mobin H, Zhang E, Bizzo BC, Mohseni I, Digumarthy SR, Kalra MK. CT Radiomics, Radiologists and Clinical Information in Predicting Outcome of Patients with COVID-19 Pneumonia. *Radiol Cardiothorac Imaging*. 2020 Jul 23;**2**(4):e200322. doi:10.1148/ryct.2020200322

16. Cai Q, Du S-Y, Gao S, Huang G-L, Zhang Z, Li S, Wang X, Li P-L, Lv P, Hou G, Zhang L-N. A model based on CT radiomic features for predicting RT-PCR becoming negative in coronavirus disease 2019 (COVID-19) patients. *BMC Med Imaging*. 2020 Oct 20;**20**. doi:10.1186/s12880-020-00521-z PMID: 33081700 PMCID: PMC7573533
